# Temporal and Habitat Dynamics of Soil Fungal Diversity in Gravel-Sand Mulching Watermelon Fields in the Semi-Arid Loess Plateau of China

**DOI:** 10.1128/spectrum.03150-22

**Published:** 2023-05-04

**Authors:** Wenqing Zhou, Xin Zhou, Lei Cai, Qi Jiang, Rong Zhang

**Affiliations:** a Institute of Plant Protection, Ningxia Academy of Agricultural and Forestry Sciences, Yinchuan, People’s Republic of China; b State Key Laboratory of Mycology, Institute of Microbiology, Chinese Academy of Sciences, Beijing, People’s Republic of China; c Institute of Forestry and Grassland Ecology, Ningxia Academy of Agricultural and Forestry Sciences, Yinchuan, People’s Republic of China; North-West University

**Keywords:** *Fusarium* wilt disease, soil fungal diversity, continuous cropping obstacle, gravel-sand mulch, sustainable agriculture

## Abstract

Mulching is an important agricultural management tool for increasing watermelon productivity and land-use efficiency because it helps improve water use efficiency and reduce soil erosion. However, there is relatively little available information regarding the effects of long-term continuous monoculture farming on soil fungal communities and related fungal pathogens in arid and semiarid regions. In this study, we characterized the fungal communities of four treatment groups, including gravel-sand-mulched farmland, gravel-sand-mulched grassland, fallow gravel-sand-mulched grassland, and native grassland, using amplicon sequencing. Our results revealed that the soil fungal communities differed significantly between mulched farmland and mulched grassland as well as the fallow mulched grassland. Gravel-sand mulch significantly impaired the diversity and composition of soil fungal communities. Soil fungal communities were more sensitive to gravel-sand mulch in grassland than in other habitats. Long-term continuous monoculture (more than 10 years) led to decreased abundance of Fusarium species, which contains include agronomically important plant pathogens. In the gravel-mulched cropland, some *Penicillium* and *Mortierella* fungi were significantly enriched with increasing mulch duration, suggesting potential beneficial properties of those fungi that could be applied to disease control. We also found that long-term gravel mulching in continuous monoculture farming could potentially form disease-suppressive soils and alter soil microbial biodiversity and fertility. Our study provides insights into the exploration of novel agricultural management strategies along with continuous monoculture practice to control watermelon wilt disease by maintaining a more sustainable and healthier soil environment.

**IMPORTANCE** Gravel-sand mulching is a traditional agricultural practice in arid and semiarid regions, providing a surface barrier for soil and water conservation. However, application of such practice in monocropping systems may lead to outbreaks of several devastating plant diseases, such as watermelon Fusarium wilt. Our results with amplicon sequencing suggest that soil fungal communities differ significantly between mulched farmland and mulched grassland and are more sensitive to gravel-sand mulch in grassland. Under continuous monoculture regimens, long-term gravel mulch is not necessarily detrimental and may result in decreased Fusarium abundance. However, some known beneficial soil fungi may be enriched in the gravel-mulch cropland as mulch duration increases. A possible explanation for the reduction in Fusarium abundance may be the formation of disease-suppressive soils. This study provides insight into the need to explore alternative strategies using beneficial microbes for sustainable watermelon wilt control in continuous monocropping system.

## INTRODUCTION

Watermelon, an important vegetable crop in the cucurbit family, requires adequate irrigation and high soil moisture for good yields. It is grown in almost all regions of the world, with global production of approximately 95 million metric tons, of which China is the world’s biggest supplier, accounting for about 75% of world production, followed by Iran, Turkey, Brazil, and the United States (1). In semiarid areas, crop growth can be severely limited by irrigation or precipitation. On the Loess Plateau, water resources are limited, and precipitation is the main source of water for crops, leading to significant drought stress. However, sufficient soil moisture can be achieved through surface mulching and other soil management practices. Gravel-sand mulching, a traditional agricultural practice for water conservation, has been used in arid and semiarid regions in the Loess Plateau in China for several centuries, where the annual mean precipitation is less than 350 mm and annual pan evaporation is greater than 1,500 mm (2). Previous studies have indicated that gravel-sand mulch has a high efficacy in increasing soil temperature and moisture, inhibiting evaporation, improving water use efficiency, and reducing soil erosion (2–4). This practice has been widely adopted due to limited water availability and high irrigation costs in semiarid areas. However, its inferiority has suggested that long-term mulching may lead to a decline in soil fertility.

Watermelon Fusarium wilt diseases, caused by Fusarium oxysporum f. sp. *niveum* (FON), can be quite frequent and severe, greatly limiting watermelon quality and yield (1). Monoculture has short-term benefits, including greater economic profits and reduced labor costs. The soil fungal community typically is one of the pivotal factors for continuous cropping of gravel-sand-mulched watermelon. However, the response of the soil fungal community to the duration of planting and subsequent replant disease of watermelon has not yet been thoroughly studied.

Soil contains a rich bank of potentially beneficial microbes; soil fungal communities play an important role in disease control and in soil nutrient and organic matter transformation (5, 6). Previous studies have shown that plant-associated soil microbiotas can enhance disease suppression and are thus used for pathogen suppression and disease control (7, 8). For example, many plant-associated fungi have antagonistic activities against Fusarium oxysporum and have been used as biological control agents, including *Clonostachys* spp., *Chaetomium* spp., *Penicillium* spp., *Talaromyces* spp., *Trichoderma* spp., and *Gliocladium* spp. (9–11). Disease outbreaks can be affected by the composition and assembly of the plant-associated microbiota as well as the concentration of pathogens. It is unclear, however, whether monoculture duration may affect soil fungal communities and watermelon-related pathogens in both gravel-sand-mulched farmland and gravel-sand-mulched grassland.

The composition and structure of fungal communities may be affected by plant species and agricultural practices, such as monoculture cultivation and water and fertilizer management (12). For example, when different crops are introduced, dynamic changes in planting years could lead to corresponding shifts in the diversity and community structure of soil fungal assemblages (13, 14). In this study, we investigated two types of gravel-sand-mulched watermelon fields in sandy wasteland in Ningxia, China. The fungal communities of watermelon fields in fields that had undergone different numbers of years of mulching-monocropping in two types of habitats (1, 5, 10, 16, and 21 years for the gravel-mulched grasslands, designated GMG1Y, GMG 5Y, GMG10Y, GMG16Y, and GMG21Y, and 1, 5, 10, 14, 23, and 24 years for the gravel-mulched farmlands, designated GMF1Y, GMF5Y, GMF10Y, GMF14Y, GMF23Y, and GMF24Y) together with native grassland (NG) and fallow gravel-mulched grassland (1 and 5 years of mulching, designated FGMG1Y and FGMG5Y) as the control treatment groups, were thoroughly examined. The main objectives of this study were (i) to identify the effect of increasing numbers of mulching years on the diversity and composition of the fungal community assembly in the monocropping system and (ii) to determine the differences in fungal diversity and composition between gravel-sand-mulched farmland and gravel-sand-mulched grassland in relation to watermelon cultivation soil. The results can be used as scientific evidence of the impact of ground mulch on soil health and provide a reasonable basis for maintaining sustainable crop production in semiarid farmlands.

## RESULTS

Sequences were preprocessed through rigorous quality control and thereafter normalized to 37,520 sequences for each sample, resulting in a total of 2,183 operational taxonomic units (OTUs). The internal transcribed spacer 1 (ITS1) region of mitochondrial DNA was used in amplicon pyrosequencing studies. A total of 5,193,513 reads were obtained, with 5,089,849 reads exceeding the quality filters. The maximum and minimum values of readings per sample reached 78,124 and 54,267, respectively. Differences in soil fungal community composition and diversity between the four habitat types were compared and analyzed.

Differences in soil fungal diversity and functional diversity of core microbiomes were assessed using pairwise comparisons between four treatment groups. Further investigation of the temporal effect of gravel-sand mulching on soil fungal communities and associated disease incidence was carried out to assess the potential risk of watermelon Fusarium wilt outbreak in a monoculture system.

### Alpha and beta diversity of the soil fungal communities in gravel-sand-mulched fields.

**FIG 1:**
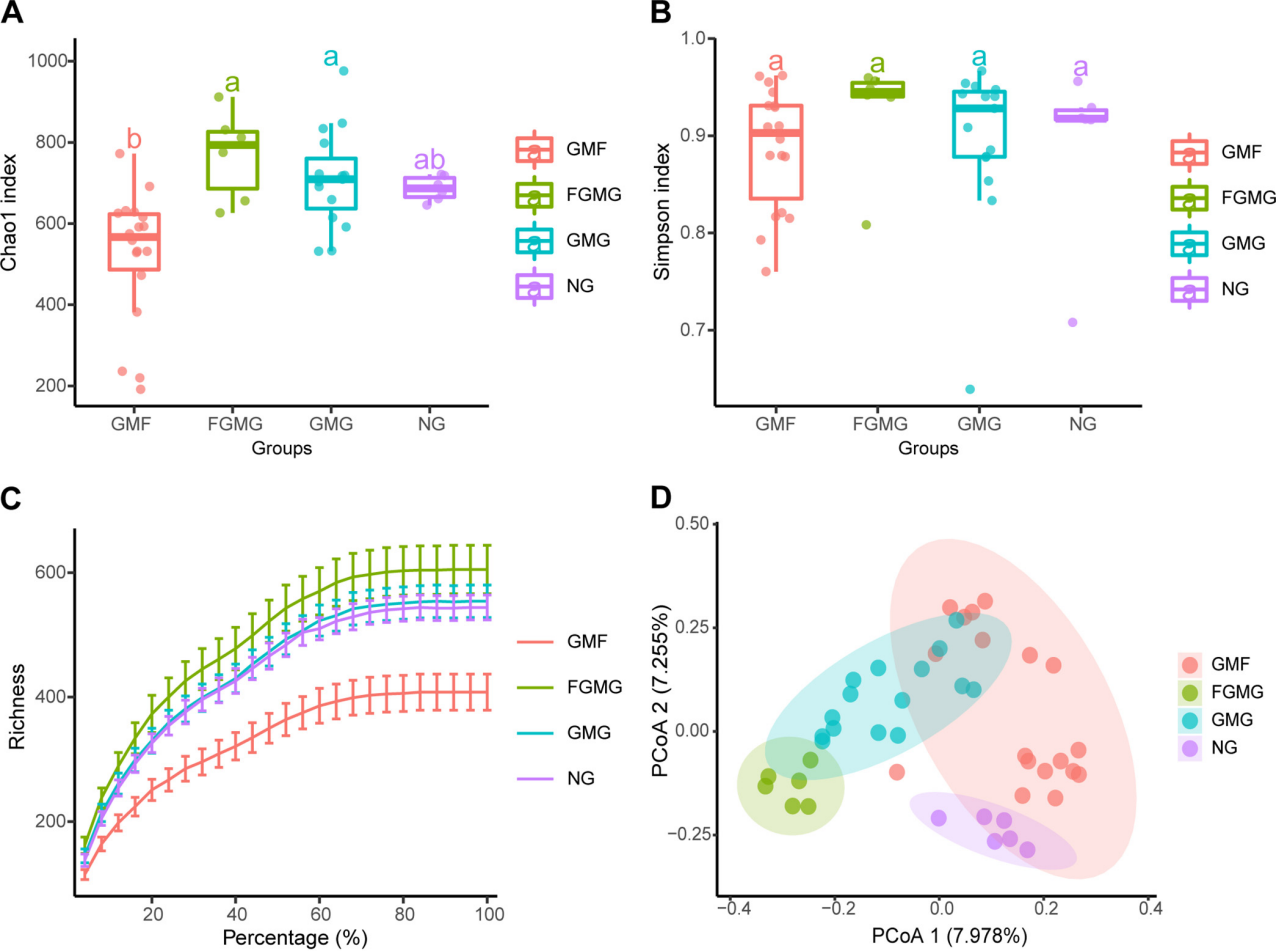
The type of habitat affects soil fungal diversities at gravel-sand-mulched field sites. (A) The Chao1 index represents fungal richness in the community. (B) The Simpson index represents species diversity in the community. (C) The rarefaction curve evaluates species richness from the sampling results at 97% similarity. Each vertical bar represents 1 standard error from the mean. Different lowercase letters indicate significant differences (*P < *0.05) between treatments. (D) Beta diversity was analyzed using PCoA ordinations of Bray-Curtis similarities calculated based on relative OTU abundances of the four different habitat groups. GMF, gravel-sand-mulched farmland; FGMG, fallow gravel-sand-mulched grassland; GMG, gravel-sand-mulched grassland; NG, native grassland.

We analyzed the alpha and beta diversity of the soil fungal communities of the four treatment groups and compared diversity differences between treatment groups. The rarefaction curves of detected soil fungal OTUs reached the saturation stage, indicating that the study captured the majority of soil fungi from each treatment. Sequencing depths were adequate to characterize fungal populations and provided sufficient coverage of the communities in the results, even after rarefaction to the lowest sequencing depth (Fig. 1C). Differences in species richness and abundance were compared between treatments by calculating Chao1 (Fig. 1A) and Simpson indices (Fig. 1B). The richness of soil fungi in the GMF sites was significantly lower than that in the GMG and FGMG sites (*P < *0.05, Kruskal-Wallis test) but slightly lower than but not statistically different from that in the NG site (Fig. 1A). The Simpson index (Fig. 1B) suggested that there was no difference in species diversity among the four treatment groups.

Principal-coordinate analysis (PCoA) revealed a significant variation in the beta diversity between treatments (Tukey’s honestly significant difference [HSD] test, *P < *0.05) (Fig. 1D). There was some similarity between the soil fungal composition in the GMG fields and the FGMG fields, as well as between the GMF fields and the NG fields. However, the most distinct differences in community composition were observed between the GMG and GMF fields. Pairwise comparisons of GMG-GMF and GMG-FGMG revealed similarities in fungal community composition. In addition, the fungal community structure of the NG fields was quite distinct from that of both the GMG fields and the FGMG fields.

**FIG 2:**
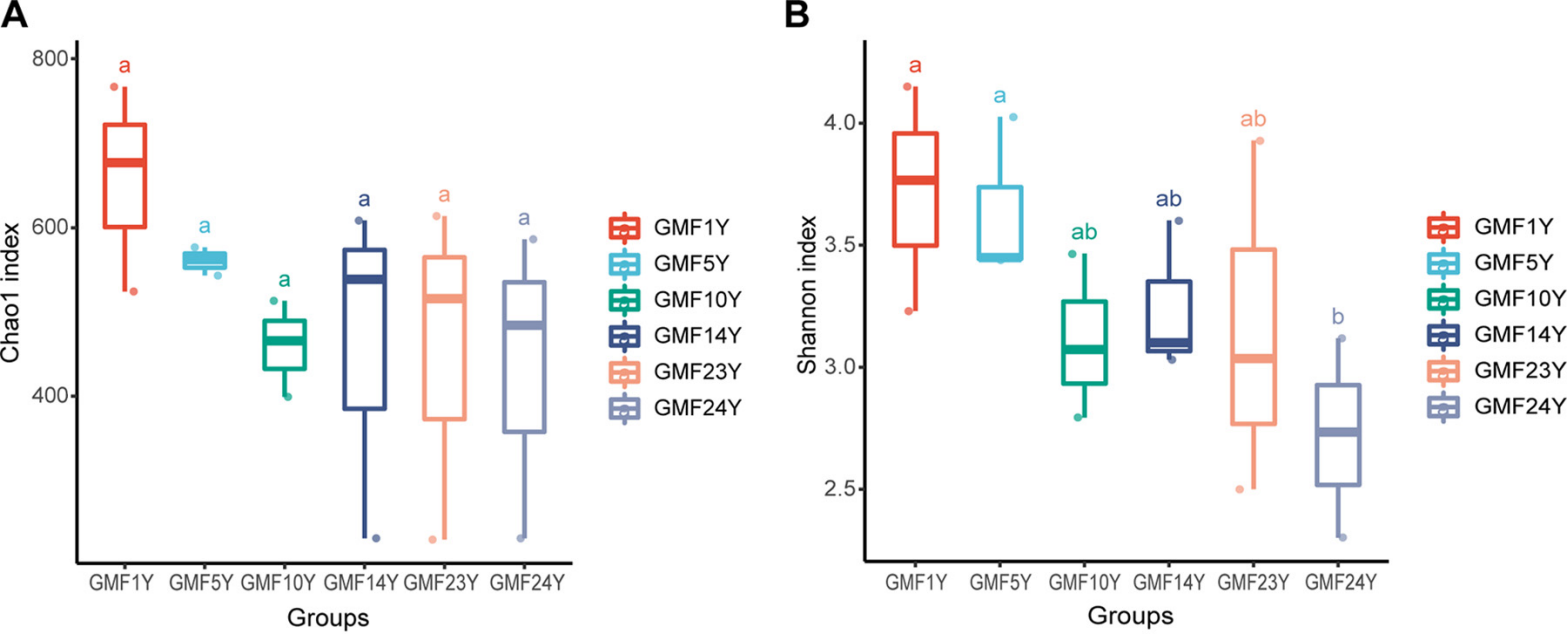
Soil fungal community richness and diversity estimates based on the Chao1 index (A) and Shannon index (B) for samples from gravel-sand-mulched farmland (GMF) fields that had undergone various numbers of years of mulching (1Y to 24Y). Different lowercase letters indicate significant differences (*P < *0.05) between treatments.

Alpha diversity analysis with pairwise comparison was performed across different numbers of years of mulching in the GMF group. Statistical analysis showed no overall difference in Chao1 richness between all treatment groups (Fig. 2A). However, soil fungal abundance showed a decreasing trend in the gravel-mulched farmland with the increase in years of mulching. A trend of decreasing species diversity with increasing number of mulching years was found, although the Shannon index did not vary significantly across the six treatment subgroups (Fig. 2B). Species diversity decreased significantly and reached the lowest level in the GMF treatment group after 24 years of mulching.

**FIG 3:**
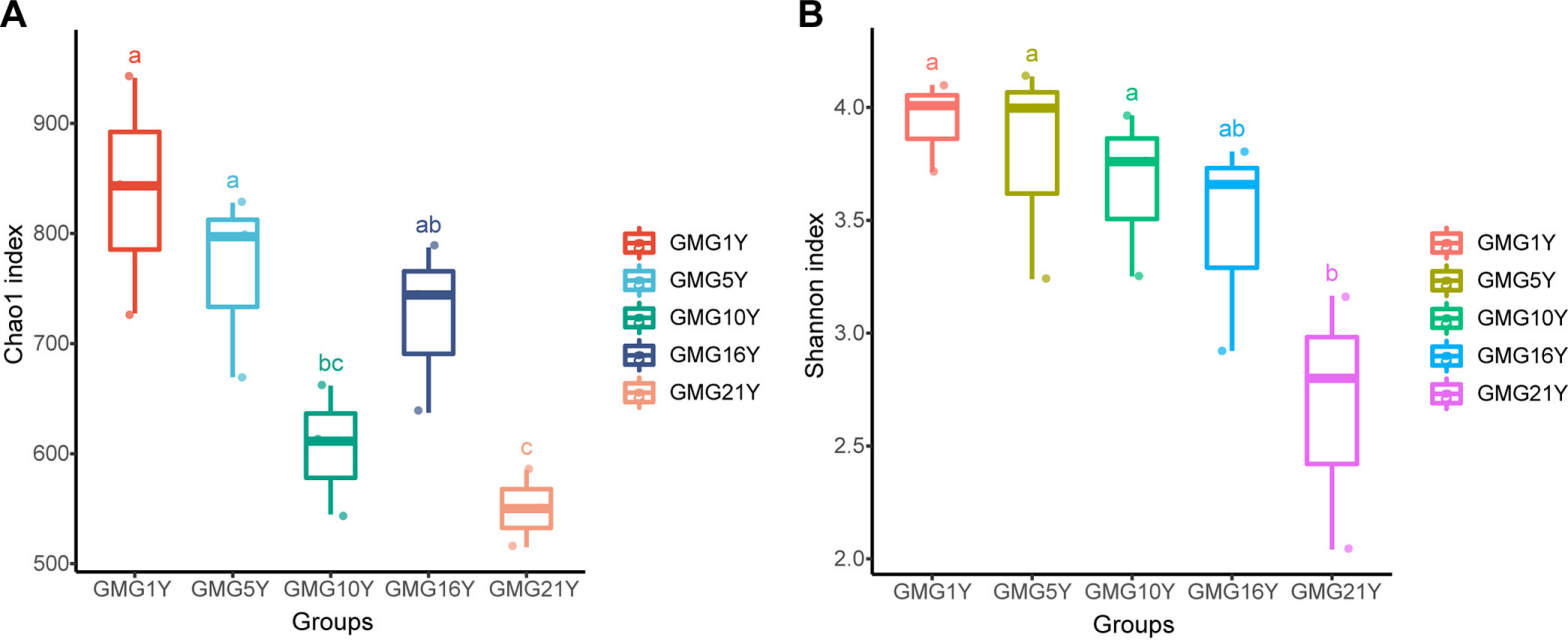
Soil fungal community richness and diversity estimates based on the Chao1 index (A) and Shannon index (B) for samples from the gravel-sand-mulched grassland (GMG) field with various numbers of years of mulching (1Y to 21Y). Different lowercase letters indicate significant differences (*P < *0.05, Kruskal-Wallis analysis) between treatments.

In the gravel-sand-mulched grassland plots, alpha diversity was also analyzed between soils that had undergone different numbers of years of mulching, ranging from 1 year (GMG1Y) to 21 years (GMG21Y). Soil fungal species richness decreased as the number of mulch years increased (Fig. 3A). The GMG1Y and the GMG21Y group had the highest and the lowest species richness, respectively, among all treatment subgroups. In the mulched grassland system, a slight decrease in species richness was observed as the number of years of mulch application increased from 1 to 21 (Fig. 3B). Soil fungal community reached the lowest diversity in the field site that had been mulched for 21 years (GMG21Y), and this diversity was significantly lower than that of the GMG1Y, GMG5Y, and GMG10Y subgroups.

### Soil fungal community assembly and species richness vary among mulch systems.

**FIG 4:**
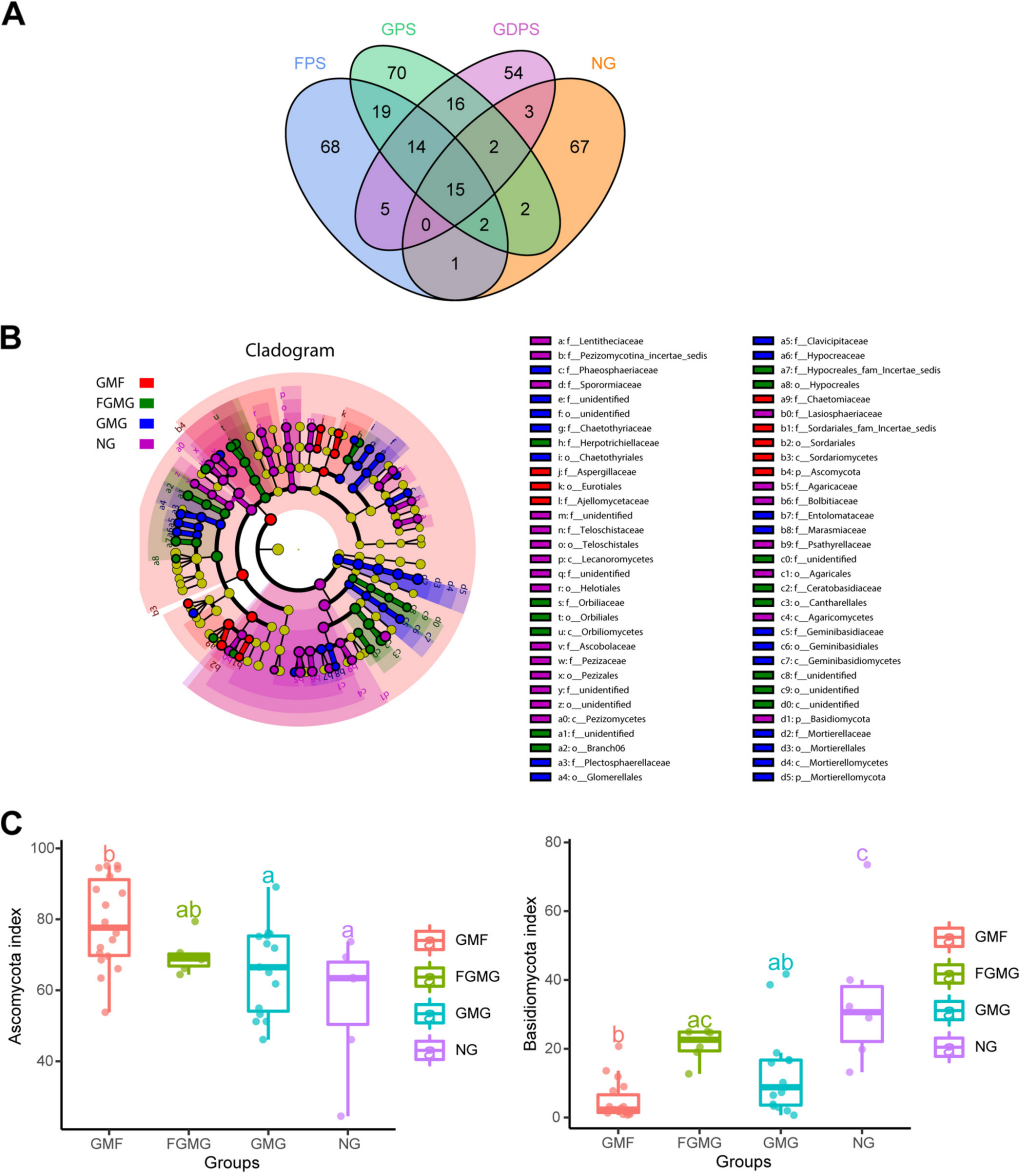
(A) Venn diagram showing shared and unique OTUs at 97% identity in the soil fungal community among the four treatment groups. Different groups are shown in different colors. The numbers represent the numbers of species shared by all groups in the overlapping area. (B) Taxonomic cladogram from LEfSe representing statistically significant differences in fungal clades among the four treatment groups. Small circles represent abundances of taxa in the respective group. Each circle’s diameter is proportional to the taxon’s abundance. (C) Relative abundances of the Ascomycota and Basidiomycota of the four treatment groups. Different lowercase letters indicate significant differences (*P < *0.05) between treatments.

To identify the dominant OTUs in different treatments, both the overlap and the distribution of the 338 most abundant OTUs across all samples were determined (Fig. 4A). Venn diagram analysis identified 124, 140, 109, and 92 OTUs in the GMF, GMG, FGMG, and NG groups, respectively. Fifteen core OTUs were identified as the common OTUs among the four groups at a 3% dissimilarity cutoff. In the pairwise comparison, the GMF and the GMG had the highest number of unique OTUs in common (50 OTUs). The differential microbiotas of the four treatment sets are presented based on linear discriminant analysis effect size (LEfSe) (Fig. 4B). Ascomycota and Basidiomycota were the two major fungal phyla among the four treatment groups. The relative abundances of both phyla were compared between treatments (Fig. 4C).

**FIG 5:**
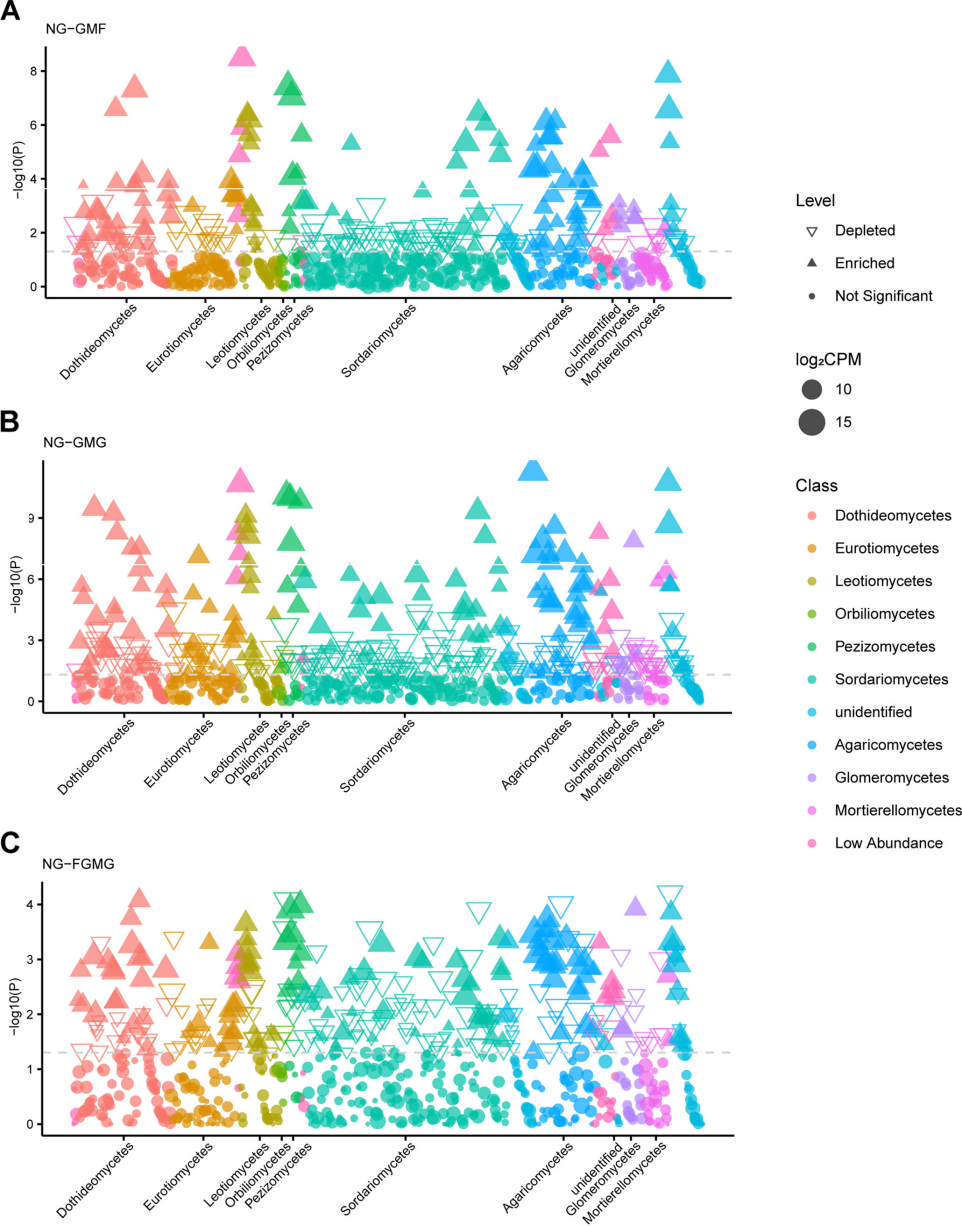
Manhattan plot showing OTUs enriched or depleted in NG group with respect to the GMF (A), GMG (B), and FGMG (C) groups. Each dot or triangle represents an individual OTU. Enriched and depleted OTUs are represented by filled and hollow triangles, respectively (false discovery rate-adjusted *P < *0.05; Wilcoxon rank-sum test). The dashed lines correspond to the false discovery rate-corrected *P* value threshold of significance (α = 0.05). OTUs are colored according to the taxonomic affiliations at the class level. The symbol size corresponds to their relative abundances in the respective samples.

Manhattan plots were used to analyze OTU enrichment based on assigned taxonomic identity in order to explore the microbiota variations of the four treatment groups at the class level (Fig. 5). The OTUs enriched in the treatment groups belonged to a variety of fungal classes (e.g., Agaricomycetes, Dothideomycetes, Eurotiomycetes, Glomeromycetes, Leotiomycetes, Orbiliomycetes, Pezizomycetes, and Sordariomycetes). A total of 127 OTUs (18.8% of total reads) were enriched in the NG group in comparison to the GMF group (Fig. 5A). In the NG groups, 199 OTUs (29.4% of total reads) and 140 OTUs (21.1% of total reads) were enriched in comparison with the GMG and FGMG groups, respectively (Fig. 5B and C).

**FIG 6:**
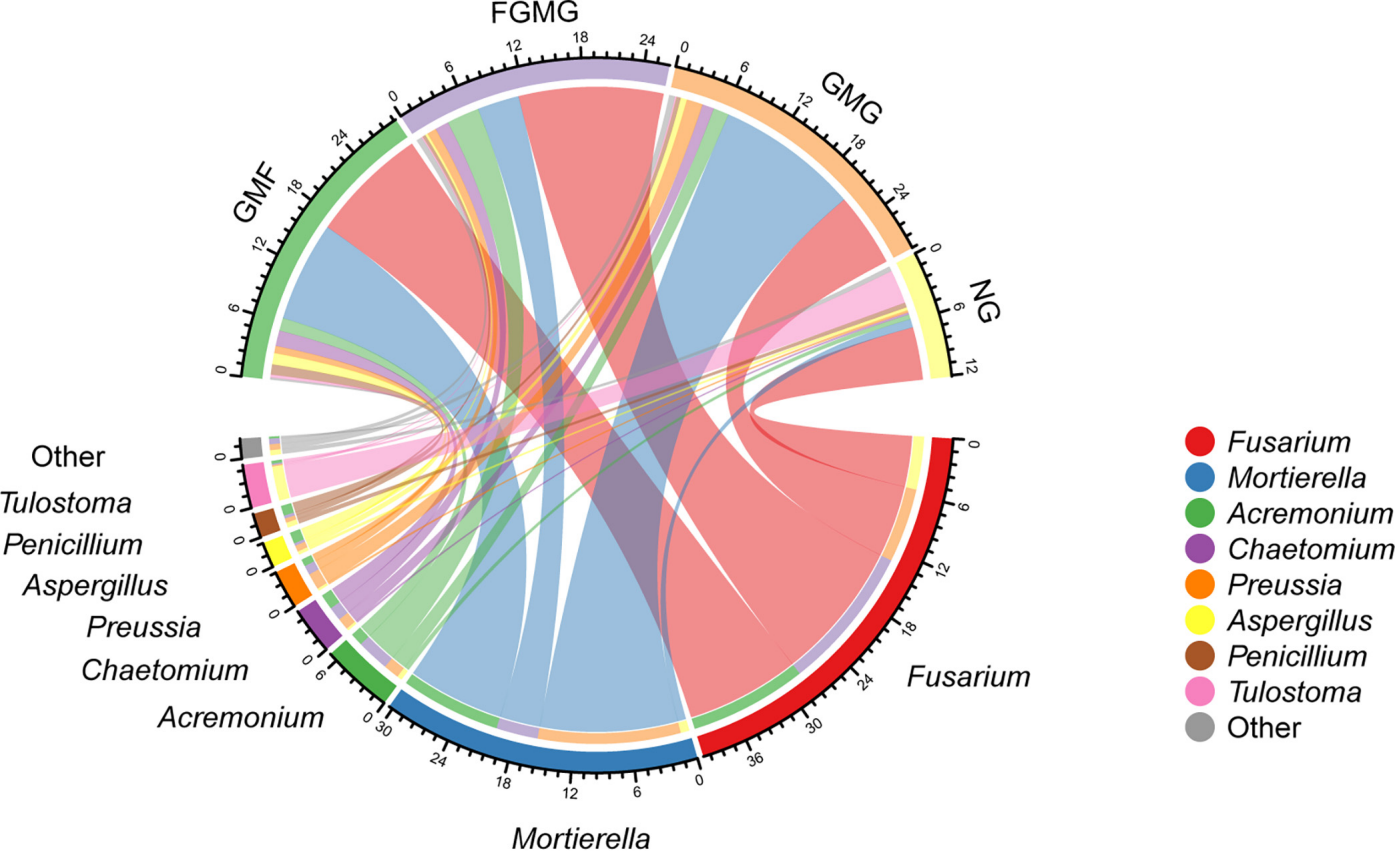
Distributions of eight core genera of soil fungi in the four treatment groups.

In addition to unidentified OTUs, a total of 291 soil fungal genera from all treatment groups were identified in this study. The distributions of eight core genera are summarized and compared in Fig. 6. Fusarium was the most abundant genus across all treatment groups, followed by *Mortierella*, *Acremonium*, *Chaetomium*, *Preussia*, *Aspergillus*, *Penicillium*, and *Tulostoma*. Fusarium dominated in the FGMG and GMF groups. *Mortierella* was more abundant in the GMG and GMF treatment groups. *Acremonium* was predominantly present in the FGMG treatment groups but not in the other three treatment groups (Fig. 6).

**FIG 7:**
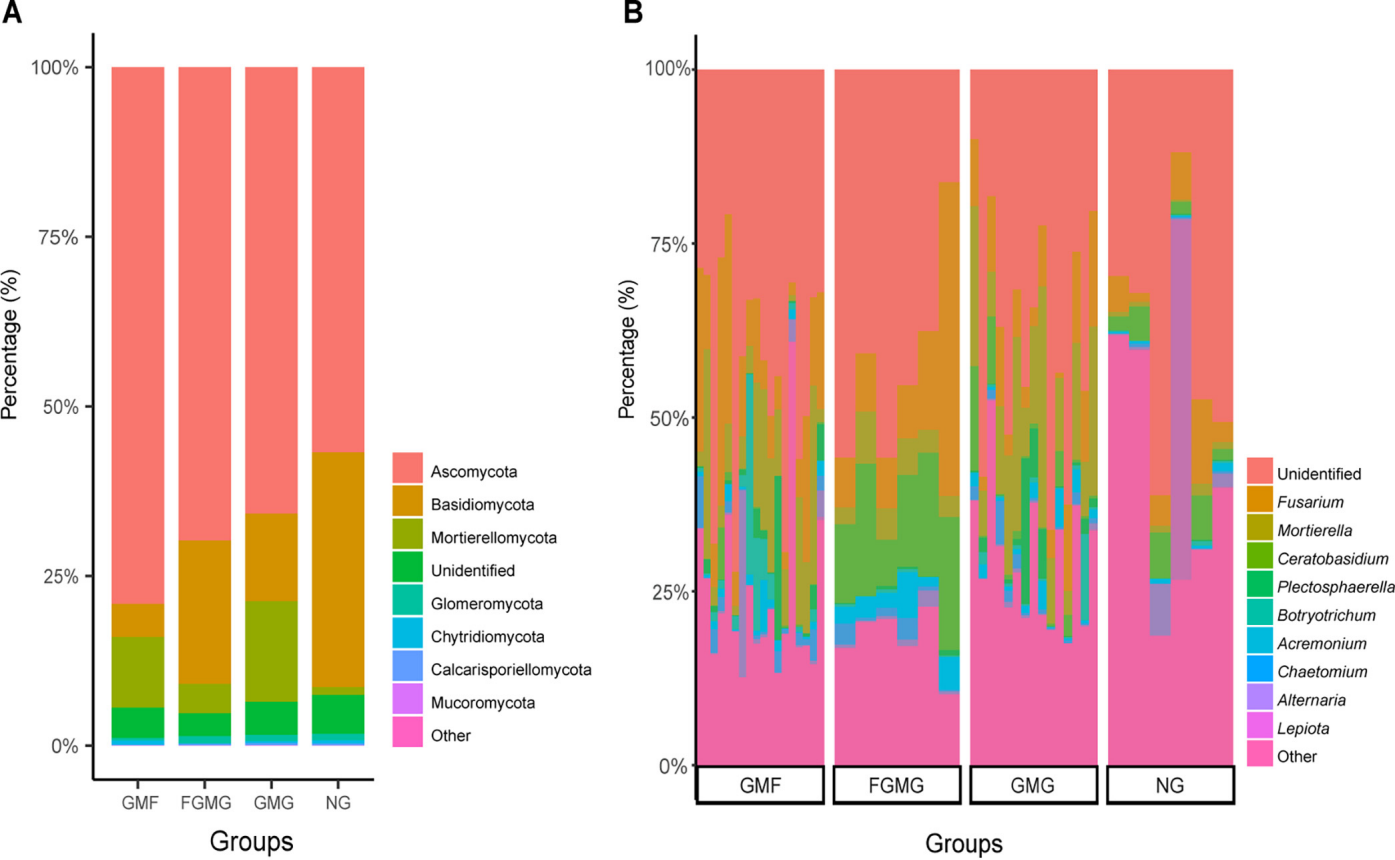
Relative abundance of soil fungi at the phylum (A) and the genus (B) levels. The community compositions are shown in treatment groups. Taxa with the relative abundance of less than 1% were categorized as “other.”

The relative abundance of the soil fungi was analyzed at both the phylum and genus levels (Fig. 7). The three most abundant phyla across the study were Ascomycota (72.1%), Basidiomycota (13.7%), and Mortierellomycota (3%) (Fig. 7A). A total of 291 fungal genera were identified across all treatment groups, with eight genera exceeding 1% relative abundance within each mulch type (Fig. 7B). The NG and GMG treatments had a relatively higher proportion of the taxa with less than 1% relative abundance at the genus level (Fig. 7B). *Ceratobasidium* was most abundant in the FGMG fields. *Mortierella* was most abundant in the GMG fields, while Fusarium was most representative of the GMF fields (Fig. 7B).

### Functional characteristics of the mycobiome among different mulch habitats and durations.

**FIG 8:**
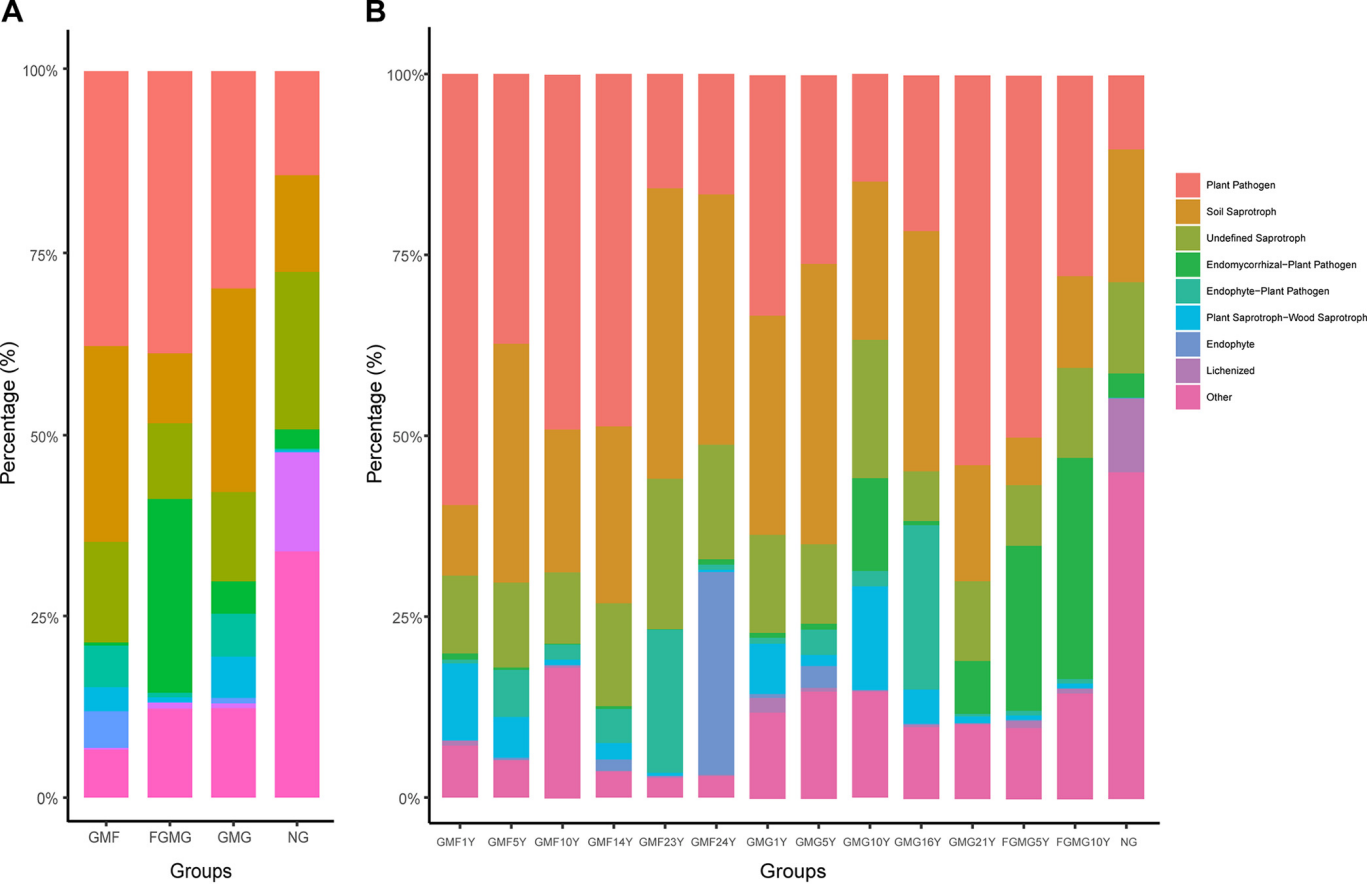
Fungal functional trait distributions of eight core genera in the GMF, FGMG, GMG, and NG groups. (A) Proportions of all ecological functional assignments within each of the four treatment groups; (B) proportions of all ecological functional assignments within each of the 14 experimental treatments. The number of years of mulching is shown as [*n*]Y.

Fungal functional traits were assigned and annotated to a total of 1,089 OTUs using the FUNGuild database (Fig. 8). The NG group contained the lowest proportion of pathogenic OTUs and the highest proportion of undefined saprotrophic and lichen OTUs in the soil fungal community (Fig. 8A). The GMG group had the largest proportion of plant saprotrophic OTUs among the treatment groups (Fig. 8A). The FGMG group contained the largest proportion of endophytic-pathogenic OTUs (Fig. 8A). The GMF group was annotated with the largest proportion of endophytic-pathogenic OTUs and few endomycorrhizal pathogenic OTUs (Fig. 8A).

The compositional structures of the ecological functions annotated with FUNGuild were compared separately across all treatment groups (Fig. 8B). The NG group showed a distinct pattern in the proportions of plant-pathogenic OTUs and the unidentified functional assignments. Plant-pathogenic OTUs were detected with the lowest proportion in the NG group, while the highest proportion of unassigned OTUs was also observed in the same treatment group (Fig. 8B). The NG group is the only one that did not contain any of the mutual endophytic and plant saprotrophic fungal functional assignments, but it had the highest proportion of lichenized fungal functional assignments. The FGMG treatment contained a higher proportion (25%) of the endomycorrhizal plant-pathogenic OTUs, which were significantly higher than in the other treatment subgroups. The proportion of the plant-pathogenic OTUs in the GMF group declined with increasing number of mulching years. In contrast, the proportions of soil pathogenic OTUs were positively correlated with the number of mulching years in the gravel-sand-mulched fields. At the GMG treatment sites, the proportions of soil pathogenic functional assignments decreased as the number of mulching years increased, as opposed to the plant-pathogenic functional assignments, which showed an ascending trend in compositional proportion over time. Grassland-derived fields appeared to contain a higher proportion of unassigned OTUs than did the GMF sites.

### Abundance of major pathogenic and beneficial fungi in different mulch groups. (i) Abundance of major pathogenic fungi in gravel-sand-mulched fields.

Soil-borne diseases often play a key role in limiting watermelon production worldwide. A recent survey of an international community of phytopathologists ranked the Fusarium oxysporum complex fifth on a list of the top 10 most important fungal plant pathogens among agriculturally and medically important fungi (1, 15). F. oxysporum is the most economically important and commonly encountered Fusarium species. The *formae speciales* of F. oxysporum are generally considered host specific (16). F. oxysporum f. sp. *niveum* (FON) has four races that infect only watermelon (17). The four races vary in their virulence and aggressiveness on watermelon cultivars (18). Due to the genetic diversity and long-term survival of this pathogen, the effectiveness of crop rotation and cultivar resistance as management strategies have been limited in the field (18).

*Alternaria* is one of the world’s most ubiquitous fungal genera, causing pre- and postharvest damage to agricultural products. Many species have been described as either saprophytes or facultative pathogens that can cause diseases with great economic impact on many agronomically important host plants, including cereals, oil crops, fruits, vegetables, and ornamentals (19, 20). *Alternaria* leaf blight is the most common disease of watermelon worldwide, major significant economic losses during the late growing season of watermelon. *Alternaria* species can produce various secondary metabolites considered plant toxins. which also cause diseases in living plants or induce health disorders in animals and humans (21).

**FIG 9:**
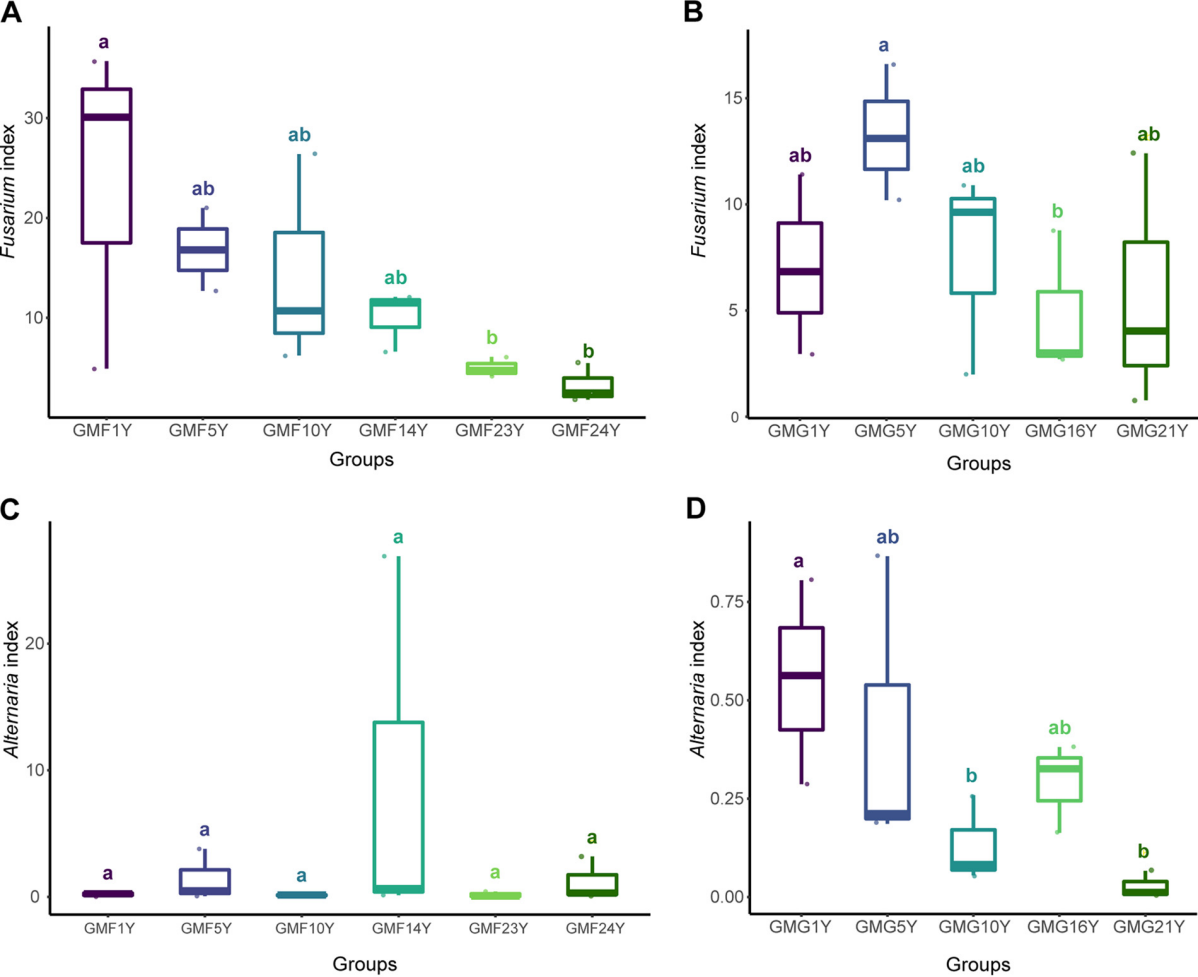
Abundance of Fusarium and *Alternaria* in GMF and GMG treatment groups. (A) Fusarium abundance in mulched farmland; (B) Fusarium abundance in mulched grassland; (C) *Alternaria* abundance in mulched farmland; (D) *Alternaria* abundance in mulched grassland. Different lowercase letters indicate significant differences (*P < *0.05, Kruskal-Wallis analysis) between treatments. The number of years of mulching is shown as [*n*]Y.

The abundances of Fusarium and *Alternaria* were analyzed and compared separately in the GMF and GMG treatment groups (Fig. 9). In the gravel-sand-mulched field, Fusarium abundance was negatively correlated with the number of mulching years (Fig. 9A). Fusarium abundance decreased significantly when the GMF field was mulched over 20 years, with the lowest Fusarium abundance reached at the 24-year GMF field site (GMF24Y; *P < *0.05, Kruskal-Wallis test). No particular trend in Fusarium abundance was found with increasing number of mulching years in the mulched grassland field (Fig. 9B). In contrast to the Fusarium community, *Alternaria* had a much lower species abundance and was barely detected in the soil microbial community in the mulched farmland and the mulched grassland (Fig. 9C and D). However, a slight decrease in *Alternaria* abundance was observed with increasing mulch duration, resulting in almost complete absence of *Alternaria* in soil after 21 consecutive years of mulching in the mulched grassland (Fig. 9D).

### (ii) Abundance of major beneficial fungi in gravel-sand-mulched fields.

In both natural and agricultural ecosystems, plants regularly interact with microorganisms, mainly bacteria and fungi. The beneficial interactions between the microbes and plants can be direct or indirect in plant nutrition, immunity, survival, and development (22). In this study, four fungal genera were selectively chosen from this group and analyzed for their abundances under different mulching conditions.

**FIG 10:**
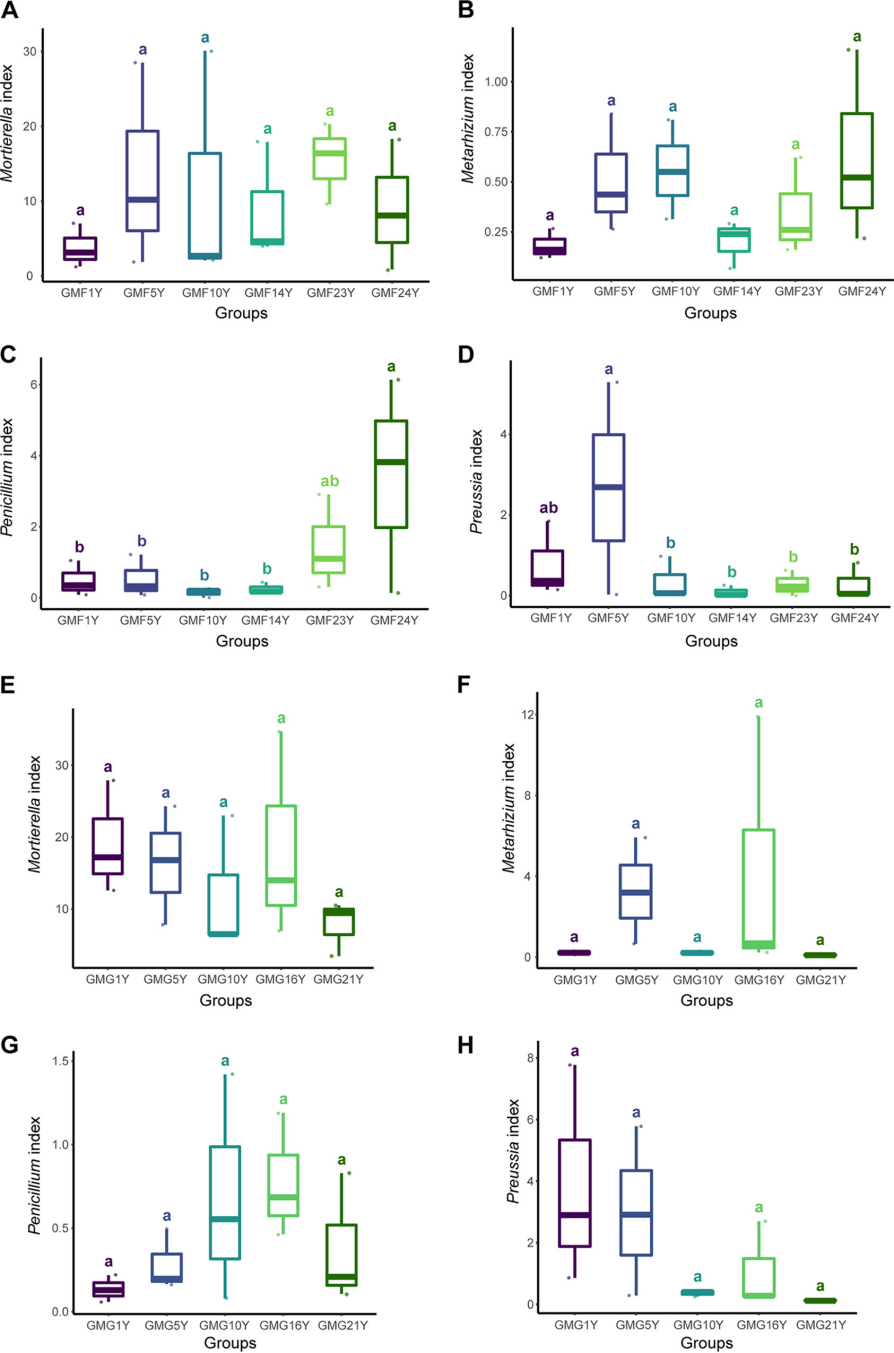
(A to D) Abundances of *Mortierella*, Metarhizium, *Penicillium*, and *Preussia*, respectively, in GMF treatment groups. (E to H) Abundances of *Mortierella*, Metarhizium, *Penicillium*, and *Preussia*, respectively, in GMG treatment groups. Different lowercase letters indicate significant differences (*P < *0.05, Kruskal-Wallis analysis) between treatments. The number of years of mulching is shown as [*n*]Y.

In the mulched farmland fields, the abundance of *Mortierella* and Metarhizium showed no statistical variation across all mulching periods (Fig. 10A and B). *Penicillium*, however, had an increasing abundance with increasing mulching years (Fig. 10C), reaching its highest level in the GMF24Y treatment (*P < *0.05, Kruskal-Wallis test). In contrast, *Preussia* abundance decreased as the number of mulching years increased (Fig. 10D). The abundance of these four genera was also examined in the mulched grassland fields. No statistical difference in fungal abundance was observed for any of the four genera (Fig. 10E to H). However, an increasing trend in the *Penicillium* abundance was observed as the mulching year increased in the mulched grassland (Fig. 10G), and an opposite trend in the *Preussia* abundance was found as mulching years increased (Fig. 10H).

## DISCUSSION

### Soil fungal community responses to mulch application in long-term continuous monoculture systems.

A number of studies have investigated the effects of gravel-sand mulch on soil microbes, soil physicochemical properties, and enzyme activities (3, 4, 23, 24). Qiu et al. (4) concluded that the fungal community was more sensitive to the gravel and straw mulch than the bacterial community after 10 years of mulch application. Zhao et al. (3) suggested that gravel-sand-mulched fields and bare land exhibited quite distinct influences on fungal community composition, and a reduced fungal diversity after long-term mulching was found in the gravel-sand-mulched fields. In our study, fungal communities responded differently to gravel-sand mulch in different ecosystems. The gravel-sand-mulched farmland had the lowest fungal alpha diversity, and it shared some similarities with the gravel-sand-mulched grassland fields according to the beta diversity measure. The soil fungal community was more sensitive to mulch application in the gravel-sand-mulched grassland than in the gravel-sand-mulched farmland in watermelon monoculture farming. In both the mulched farmland and mulched grassland fields, the alpha diversity of the soil fungal community decreased as mulch duration extended. A total of 173 depleted OTUs and 165 enriched OTUs were observed in the native grassland treatment compared to the gravel-sand-mulched grassland treatment, which showed the greatest variation in community composition with mulch application.

In a previous study of gravel-straw mulched fields, the relative abundance of Ascomycota decreased in contrast to the increased relative abundance of the Mortierellomycota and Glomeromycota as mulch duration extended, according to Qiu et al. (4). A 3-year gravel mulch experiment conducted by Lv et al. (25) suggested that the relative abundance of Ascomycota significantly increased after mulch application, correlated with decreased relative abundance of *Alternaria*, *Mortierella*, Fusarium, and *Purpureocillium*. In contrast, our study suggested that the relative abundances of Ascomycota and Basidiomycota did not vary significantly between mulching years of the same mulch type. However, Ascomycota fungi were most abundant in the mulched farmland fields and least abundant in the native grassland, in contrast to the relative abundance of Basidiomycota, which was highest in the native grassland and lowest in the mulched farmland field.

### Functional diversity of soil fungi in gravel-mulched continuous monoculture fields.

Certain Fusarium spp. and *Alternaria* spp. are among the most extensively studied fungal pathogens of watermelon due to their destructive impacts on yield and quality. However, only a few studies have discussed the variation in Fusarium abundance in gravel-mulched fields, and almost none have focused on *Alternaria* response to mulch application. Zhao et al. (3) suggested that Fusarium abundance was reduced in fields that had undergone long-term gravel-sand mulching in the absence of a crop. In our study, Fusarium abundance decreased as the number of mulching years increased in the GMF fields, in comparison with no clear correlation between the Fusarium abundance and mulch year in the GMG fields. Meanwhile, *Alternaria* abundance remained significantly lower than Fusarium abundance with no variation across mulching duration treatment in GMF fields, whereas a declining trend in the Fusarium abundance as mulching duration increased was observed in the GMG fields.

Some *Mortierella* spp. have been recognized for their ability to degrade organic pollutants for soil remediation (26, 27). Studies have indicated that *Mortierella* spp. may be related to the inhibition of soilborne disease, in particular those diseases caused by pathogenic Fusarium, by transforming phosphorus in the soil (28, 29). Some Metarhizium spp. in rhizospheres can act as both plant growth promoters and insect pathogens (30, 31). Metarhizium spp. also can mediate essential nutrient transfer to host plants (32) as well as enhancing plant tolerance to abiotic stressors (33). *Penicillium* spp. have been widely accepted as biological control agents against several phytopathogens (34, 35) or as plant growth stimulants (36). *Preussia* is ecologically diverse but not well understood. Several *Preussia* species are known to produce secondary metabolites, in particular, preussomerins with antimicrobial activities (37, 38). Al-Hosni et al. (39) suggested that some *Preussia* fungi may potentially improve crop growth and yield.

In this study, a quantitative analysis was performed to examine the abundance of *Mortierella*, Metarhizium, *Penicillium*, and *Preussia* in GMF and GMG fields over a series of mulching years ranging from 1 year to 24 years. Neither *Mortierella* nor Metarhizium showed any long-term trends in abundance dynamics under any type of mulch applications. However, increasing *Penicillium* abundance and decreasing *Preussia* abundance were observed in the GMF fields as the number of mulching years increased. Similar trends were also found in the GMG fields. No previous study has focused on discussing the responses of these four fungal groups to gravel mulch type or mulch duration.

### The main challenge of gravel-sand mulch in a continuous monoculture agroecosystem in a semiarid area.

Continuous cropping has a widely recognized negative effect on plant growth and the emergence of plant diseases. Watermelon has been grown continuously and intensively in the study area for several decades, making watermelon wilt the most common and severe plant disease in this area. In 2021, a local plant disease scouting program provided data suggesting that the severity of Fusarium wilt disease was moderate to mild, with less than 20% of farms observed to have disease symptoms. Previous studies have shown that long-term (more than 10 years) continuous cropping can create disease-suppressive soils (40, 41). In disease-suppressive soils, plants suffered less than expected from specific soilborne diseases due to the mutualistic associations between roots and specific soil microorganisms (42). Consistent with previous studies, we found that the Fusarium abundance decreased significantly with increasing duration of continuous cropping. Another possible reason for the relatively low severity and limited spread of watermelon wilt in 2021 may be the constant and extreme drought throughout the growing season. Given the fact that most of the watermelon fields in this area were equipped with drip-irrigation systems but used limited or almost no irrigation throughout the year, water scarcity can not only limit disease outbreaks but also reduce plant growth and yield.

In the Xiangshan region, a typical arid and semiarid area of the Loess Plateau in China, almost entire agricultural fields have been mulched with gravel-sand as an important water conservation technique for the past few decades. Gravel-sand mulch has been found to negatively affect the sustainability of soil organic matter and soil symbiotic microbes (43–45). Long-term continuous monoculture with gravel mulching in this particular area has already resulted in a substantial decrease in net farm income due to low yields (46) and high disease management cost (47). Earlier studies focusing on sustainable agricultural management in this region found that the ecological footprint in the Xiangshan area increased by 87.5% from 2003 to 2009, while the per capita biocapacity increased by only 63.1%, resulting in a maximum per capita ecological debt of 1.08 ha in 2009 (47). Therefore, finding alternative strategies for sustainable agricultural systems that address environmental and public health concerns has become the next major challenge for local agriculture in the Xiangshan area.

## MATERIALS AND METHODS

### Site description.

This experiment was conducted in the gravel-sand-mulched fields in the Xiangshan area (Zhongwei, Ningxia Province; 37°4′17′′N, 105°5′15′′E; elevation, approximately 1,600 m), located to the west of the Loess Plateau in northwest China in August 2021. The average annual precipitation in this area over the past decade is 320.1 mm, with an average annual evaporation of over 2,000 mm. All fields were covered by an estimated 10-cm-deep layer of 1:1 (vol/vol) gravel-sand mulch. The watermelon seedlings were manually transplanted to the field after cultivation. The majority of the fields in this area were equipped with subsurface drip irrigation systems. However, limited or no irrigation was applied throughout the growing season due to the extreme shortage of water.

### Experimental design and soil sampling.

All field sites were derived from either a grassland or a farmland habitat, being either a planted or a vacant field at the time of sampling. One native grassland site was included as the control. A total of 45 samples were obtained from 15 field sites, consisting of six gravel-sand-mulched farmland sites, five gravel-sand-mulched grassland sites, two fallow gravel-sand-mulched grassland sites, and one native grassland site as the control. The experimental design, including the number of planted or vacant years for each field site, is provided in Table 1.

**TABLE 1:** Experimental design and sample collection

Land type	Vegetation	No. of yrs mulched
GMF	Watermelon	1, 5, 10, 14, 23, 24
GMG	Watermelon	1, 5, 10, 16, 21
FGMG	Artemisia annua, Artemisia scoparia, Convolvulus tragacanthoides, Oxytropis aciphylla, Salsola ruthenica, Stipa capillata, Suaeda salsa	5, 10
NG	Salsola passerine, Reaumuria songarica, Oxytropis aciphylla	

Soil samples were collected following a randomized complete block design (RCBD), with three representative sampling points per site. The gravel-sand mulch was removed before sampling. Field soil was obtained using a soil drill and screened through a 4-mm sieve. The fresh soil was then thoroughly homogenized, and a portion was sealed in a labeled zipper bag. All samples were transferred to the laboratory and stored at −20°C for DNA analysis.

### DNA extraction, PCR amplification, library preparation, and sequencing.

Microbial genomic DNA was extracted from approximately 0.5 g soil using the Tiangen magnetic soil and stool DNA kit (DP712; Tiangen Biotech Co., Ltd., Beijing, China). DNA concentration and purity were quantified and diluted to 1 ng μL^−1^ using sterile water as the final system.

The fungal ITS1-1F region was amplified using the primer pair ITS1-1F-F/ITS1-1F-R (48) for fungal samples with barcodes. PCRs were performed in 25-μL reactions, with 15 μL Phusion high-fidelity PCR master mix (New England Biolabs, Inc., Ipswich, MA, USA), 0.2 μM forward and reverse primers, and about 10 ng template DNA. Thermal cycling consisted of initial denaturation at 98°C for 1 min, followed by 30 cycles of denaturation at 98°C for 10 s, annealing at 50°C for 30 s, and elongation at 72°C for 30 s, with a final step at 72°C for 5 min. An equal volume of 1× loading buffer (containing SYBR green) was mixed with PCR product, and the electrophoresis detection was conducted on a 2% agarose gel. PCR products were mixed in equidensity ratios and purified with a QIAEX II gel extraction kit (Qiagen, Germany). Sequencing libraries were prepared and indexed using a TruSeq DNA PCR-free sample preparation kit (Illumina, USA) following the manufacturer’s recommendations. Library concentration and size distribution were assessed with a Qubit 2.0 fluorometer (Thermo Scientific, Wilmington, DE, USA) and an Agilent 2100 Bioanalyzer. Each cell was paired-end sequenced (200-bp reads) on an Illumina NovaSeq platform.

### Sequencing data analysis.

Paired-end reads were assigned to samples based on the barcodes and truncated by cutting off the barcodes and primer sequences. FLASH (V1.2.7) (49) was used to splice the reads of each sample with a strict filtering process until the chimera sequence was removed and the final effective raw tags were obtained (50, 51). All sequences were clustered to OTUs at 97% similarity using the UPARSE algorithm in the USEARCH11 software (52). Representative sequences were selected with the highest occurrence frequency in OTUs, and species annotation analysis was performed using the BLAST method in QIIME (V1.9.1) (53) and the UNITE (V7.2) database (54). Statistical analyses were conducted on the community composition of each sample at the genus and family levels. Rarefaction was used to standardize the number of sequences of each amplicon library size obtained from sequencing (55). The abundances of OTUs were normalized using the least abundant sequences.

### Statistical analysis.

Soil microbial diversity, including the observed OTUs, Good’s coverage, and Chao1 and Shannon indices, was calculated using the vegan package in R (V4.1.2C) (56). Tukey’s HSD test was used to analyze the differences in the alpha diversity and relative abundances between treatments. Data analysis was conducted using vegan (56), pheatmap, dplyr, and other packages in the R environment. The alpha diversity (i.e., Chao1 and Simpson indices) among different treatments was analyzed using the estimateR and diversity functions in the vegan package (56). Independent one-way analysis of variance (ANOVA) followed by Tukey’s HSD test (*P < *0.05) was used for analyzing the differences in alpha diversity of different taxonomic groups that the temporal dimension or habitat types might have caused.

Based on the Bray-Curtis distance, the multiresponse permutation procedure (MRPP) was used to analyze the differences in soil fungal community structure. PCoA was performed to analyze the beta diversity of different field type treatments using the vegan package. OTUs with relative abundances greater than 0.04 in each treatment were kept for calculating the phylogenetic trees using the neighbor-joining (NJ) algorithm in MEGA X (V10.2) (57). LEfSe was employed to reveal the statistically significant differential abundances of fungal taxa corresponding to different durations of watermelon monoculture (58). The functional groups and ecological niches (guilds) of fungal taxa were annotated by comparison against the FUNGuild (https://github.com/UMNFuN/FUNGuild) database (59).

### Data availability.

Raw sequence reads were submitted to the BIG submission portal in the BioProject archive under project number PRJCA015124 (GSA accession number CRA009952).
